# Measures of spiritual and transpersonal constructs for use in yoga research

**DOI:** 10.4103/0973-6131.53837

**Published:** 2009

**Authors:** Douglas A MacDonald, Harris L Friedman

**Affiliations:** Department of Psychology, University of Detroit Mercy, USA; 1 Department of Psychology University of Florida, USA

**Keywords:** Consciousness, Indian approach, paper-pencil tests

## Abstract

This article presents information on standardized paper-and-pencil measures of spiritual and transpersonal constructs that hold promise for use in yoga research. Nine instruments are discussed at length including the Assessment Schedule for Altered States of Consciousness, Ego Grasping Orientation, Expressions of Spirituality Inventory, Hindu Religious Coping Scale, Measures of Hindu Pathways, Self-Expansiveness Level Form, Spiritual Orientation Inventory, Spiritual Transcendence Scale, and the Vedic Personality Inventory. As well, a listing of an additional 14 measures, along with primary citations, is provided. In conclusion, the authors proffer recommendations for the use of psychometric tests and provide a general proposal for programmatic research.

## INTRODUCTION

Over the past three decades, there has been an increasing interest within the scientific community in concepts of spirituality and methods of spiritual transformation such as yoga, as well as in exploring their relationship and relevance to human functioning and well-being. Ostensibly accompanying this growing interest has been efforts at devising methodological approaches to their study that do justice to their subtlety and complexity. Unfortunately, there is little consensus as to how such concepts should be best defined and investigated. This is most evident in the ongoing debate within transpersonal psychology, the subdiscipline of psychology most concerned with such concepts and practices, regarding the centrality of qualitative (e.g., phenomenological) versus quantitative (e.g., standardized testing) methods, which we have discussed previously in another article.[1]

Notwithstanding such controversy, perusal of the available literature within the quantitative domain reveals a broad array of measures that operationally define a variety of concepts falling within the domain of spirituality and transpersonal psychology. Although we have also argued elsewhere[2] that such measures have inherent limitations and should not be seen as the sole or necessarily best means for studying spiritual and transpersonal, as well as transcendent, concepts, and practices, they nonetheless hold potential to help systematically standardize and organize research activities and findings to better facilitate the integration of research into the corpus of mainstream scientific knowledge. As such, we maintain that psychometric tests should be seen as an ally and not an enemy of investigators interested in the study of spiritual and transpersonal concepts and practices. In this vein, the purpose of this article is to provide information about available assessment instruments that appear promising for use by yoga researchers and/or transpersonally oriented studies involving Indian samples.

In total, nine measures are presented at length [Table 1a–c], and an additional 14 are listed in Table 2. Based upon our previous test survey publications[2–4] as well as a perusal of literature published from 1999 to present through a variety of online databases (e.g., PubMed, PsycINFO), we identified several instruments that either measure a construct of central importance to spiritual and transpersonal studies (e.g., altered states of consciousness, spirituality, methods of transformation, etc.) and/or have been utilized in at least one cross-cultural study. Because of space limitations, we arbitrarily selected only nine of the tests that we thought would be of greatest interest to readers, who are invited to look at our previous works for a survey of more than a hundred measures.

**Table 1a:** Summary of key information about tests presented

	Assessment schedule for altered states of for consciousness[5]	Ego-grasping orientation[11]	Expressions of spirituality inventory[13,14]
Construct(s) assessed	Altered states experiences	Ego grasping orientation	Spirituality
Number of items	325	20 (Note- 30 item version is available)	98
Subscales	14	None	5
Response	Five-point Likert scale	True/False	Five-point Likert scale
Format	Likert scale		Likert scale
Time to administer	60-90 minutes total (Note- subscales can be used separately)	10 minutes	20-40 minutes (Note- 30 item takes about 10-20 minutes)
Reliability/ Validity	Good interitem consistency; Evidence of adequate content, criterion, discriminant, and factorial validity	Good interitem consistency and test-retest reliability; Evidence of adequate criterion, convergent and discriminant validity	Both 98- and 30- item versions show good interitem consistency; Evidence of adequate criterion, convergent, discriminant, and factorial validity
Availability	From author-contact R. vanQuekelberghe, Universitat Koblenz-Landau; Fachbereich 8 Psychologie; Im Fort 7 6740 Landau, Germany	From journal article[11]	From author-contact Dr. D. A. MacDonald, University of Detroit Mercy, Department of Psychology, 4001 W. McNichols Rd, Detroit, MI 48221, macdonda@udmercy.edu
Cost	None	None	None
Special notes	ASASC designed to be a comprehensive measure of altered states	EGO designed to measure Taoist orientation to ego functioning	ESI designed to serve as a comprehensive measure of spirituality. Has been used cross-culturally

EGO - Ego grasping orientation, ASASC - Assessment schedule for altered states of consciousness, ESI - Expressions spirituality inventory

**Table 1b:** Summary of key information about tests presented

	Hindu religious coping scale[16]	Measures of hindu pathways[17]	Self-expansiveness level form[18]
Construct assessed	Hindu religious coping strategies	Hindu religiosity as manifest in daily living	Self-expansiveness
Number of items	20	37 (in original form) (16 in revised form)	18
Subscales	Three	Four	Three
Response format	Four-point scale	Varies across subscales	Five-point Likert scale
Time to administer	10-15 minutes	10-20 minutes	5-10 minutes
Reliability/Validity	Good interitem consistency; Evidence of adequate convergent, discriminant and criterion validity	Good interitem consistency; Some support for discriminant and criterion validity	Good interitem consistency; Evidence of adequate factorial, criterion, and discriminant validity
Availability	From journal article[16]	From journal article[17]	From journal article[18]
Cost	None	None	None
Special notes		Special notes The SELF has been used cross-culturally (India)	

**Table 1c:** Summary of key information about tests presented

	Spiritual orientation inventory[21]	Spiritual transcendence scale[23]	Vedic personality inventory[26,14]
Construct assessed	Spirituality	Spirituality	Personality (Gunas)
Number of items	85	24	56
Subscales	Nine	Three	Three
Response format	Seven-point Likert scale	Five-point Likert scale	Six-point Likert scale
Time to administer	20-40 minutes	5-20 minutes	15-30 minutes
Reliability/Validity	Good interitem consistency; Evidence of adequate content and criterion validity	Good interitem consistency; Evidence of adequate factorial, criterion, convergent and discriminant validity	Evidence of adequate factorial, convergent, discriminant, and criterion validity
Availability from journal	Contact: Sara Elkins 33442 Cape Bay Place Dana Point, CA 92629	From journal article[23]	From journal article[26]
Cost	$1 US per copy (Note- price subject to change)	None	None
Special notes	The SOI is a measure of humanistic spirituality. It has been used cross-culturally (India)	The STS has been used cross-culturally (India)	The VPI is based upon Vedic literature and has been used for Yoga studies with Indian sample[33]

**Table 2:** Some additional measures of potential interest to investigators doing yoga, spiritual and/or transpersonal research

Name of test	Construct(s) assessed
Boundary questionnaire[34]	Psychological boundary thickness/thinness defined across 12 domains including different states of consciousness/perception and different levels of social identity
Dimensions of meditative experience[35]	Multi-dimensional measure of the phenomenology of meditative experience
East-West questionnaire[36]	Eastern versus western worldviews as defined across 5 areas including man and the spiritual, man and nature, man and society, man and himself, and the rationality of man
Ego permissiveness inventory[37,38]	Ego permissiveness (aka openness) as operationalized across nine dimensions
Feelings, reactions and beliefs survey[39,40]	Nine aspects of personality as defined in rogerian theory
Immanence scale[41]	Immanence defined as present-centeredness, motivation toward transcendence, and acceptance as response to threats
Paranormal beliefs scale[42,43]	Seven dimensional model of paranormal beliefs
Perceived wellness survey[44]	Six dimensional model of holistic well-being
Phenomenology of consciousness inventory[45,46]	Twelve dimensions of phenomenological experience associated with different stimulus conditions
Psychomatrix spirituality inventory[47]	Seven dimensional model of spirituality defined in terms of experience and practices
Scales of psychological well-being[48,49]	Multidimensional model of eudiamonic well-being
Spiritual well-being scale[50,51]	Two-dimensional model of spiritual well-being (religious well-being; existential well-being)
Temperament and character inventory[52]	Seven factor measure of personality which includes a self-transcendence dimension
Transliminality scale[53,54]	Unidimensional measure of psychological boundary permeability

### Assessment schedule for altered states of consciousness

The assessment schedule for altered states of consciousness (ASASC) was developed by VanQuekelberghe, Altstotter-Gleich, and Hertweck[5] to serve as a comprehensive measure of altered or nonordinary states of consciousness. The construction of the test was guided by extant taxonomies and definitions of altered states offered by several seminal writers in the area including Fischer,[6] Gowan,[7] Tart,[8,9] and most importantly Ludwig.[10]

The instrument is made up of 325 items which are unevenly divided across 14 subscales. The subscales all utilize the same five-point response scale (ranging from 0 = not at all to 4 = completely) and each has its own instructions so that they can be used separately if desired. The subscales are as follows: Personal data (i.e., demographics and background information about behaviors associated with nonordinary states of consciousness such as drug use, meditation, and psychotherapy), Extraordinary Mental Processes (i.e., unusual thought patterns, strange ideas, highly unusual experiences), Parapsychology—Own Experiences (i.e., personal experiences of parapsychological phenomena), Parapsychology—Own View (i.e., estimates of the probability of occurrence of parapsychological events), Esoterics (i.e., engaging in practices associated with metaphysical and paranormal phenomena such as astrology, séances, spiritual healing), Positive Mystical Experiences (i.e., ecstatic states of consciousness similar to peak experiences), Negative Mystical Experiences (i.e., extreme negative experiences such as feeling surrounded by evil forces), Imagination (i.e., imagination and visualization ability), Dreams (i.e., preoccupation with dreams and frequency of occurrence of different types of dreams), Dissociation (i.e., tendencies toward trance-like and dissociative states), Hallucinations (i.e., images and mental processes generally associated with psychopathological hallucinatory states), Hypersensitiveness (i.e., heightened body sensitivity, synesthetic experiences), Changed Feeling of Time and Space, and Change (i.e., long term effects of nonordinary states on personal functioning and development).

The psychometric properties of the ASASC subscale scores appear to be generally satisfactory as reflected in good interitem reliability coefficients, favorable factor analytic and multidimensional scaling findings, and theoretically expected subscale intercorrelations. The instrument also appears to be unaffected by a variety of demographic variables including age, education, and religious denomination. Finally, the ASASC has been used to develop score profiles for discrete groups of respondents including those who have had drug experiences, people who engage in esoteric practices, and people suffering from a variety of forms of psychopathology including heroine addiction, major depression, and schizophrenia—as documented in one of our review articles.[2]

### Ego grasping orientation

Knoblauch and Falconer[11] based the ego grasping orientation (EGO) upon the adaptation of the Taoist concepts of *yin-yang, wu-wei,* andteto psychotherapy. The EGO is a measure of Taoist orientation that assesses the construct of ego grasping, defined by Knoblauch[12] as “a dualistic stance that is marked by the person’s attempts to make things more positive while striving to eliminate the negative aspects of human experience” (p. 55). Knoblauch argued that a person high in ego grasping would be seen from a Taoist perspective as being egocentric and prone to egoic idealism.

Items for the EGO were developed based upon statements made by clients in psychotherapy that were perceived by the test authors as supporting the notion of ego grasping. The instrument is made up of 20 true/false self-descriptive statements. Respondents are asked to indicate whether or not each of the statements is true in describing their beliefs and behavior. Scoring of the instrument involves reverse scoring negative items and then summing items responded to in the direction of ego grasping. Higher EGO scores are associated with higher levels of ego grasping. The EGO has been found with North American samples to produce scores that demonstrate satisfactory reliability (both test-retest and interitem) and good convergent, discriminant, and criterion validity, as referenced in one of our review articles.[2]

### Expressions of spirituality inventory

MacDonald[13,14] designed the expressions of spirituality inventory (ESI) to measure a multidimensional factor analytic model of spirituality. The motivation for the development of the ESI was derived from its author’s observation that there was a need for an empirically grounded structural framework for organizing the burgeoning number of measures and conceptualizations of spirituality that were appearing (and continue to appear) in the literature. Based upon the factor analyses of a total of 19 different extant measures of spirituality and related concepts, as well as an original item pool, with data obtained from approximately 1400 Canadian university students, MacDonald identified five factors that seem to comprise major facets of spirituality. These factors are named Cognitive Orientation Toward Spirituality (i.e., beliefs about the existence and relevance of spirituality for day-to-day life), Experiential/Phenomenological Dimension (i.e., spiritual experience), Existential Well-Being (i.e., felt sense of meaning and purpose and sense of being able to cope with adversity), Paranormal Beliefs (i.e. belief in the existence of parapsychological phenomena), and Religiousness (i.e., intrinsic religious orientation, includes religious practices such as prayer and meditation). Using items from his original item pool, MacDonald developed a 98-item version of the ESI. Shortly thereafter, he constructed a shorter 30-item version using items from the longer test. The test uses a five-point response scale that has respondents rate the extent to which they agree with the items. Scoring entails first reverse coding negatively worded items and then summing item responses to arrive at dimension scores.

Research on the psychometric properties of both the 98- and 30-item versions of the test has shown that the ESI dimension scores have satisfactory reliability and good factorial, convergent, discriminant, and criterion validity.[4,13,14] At the time of preparing this article, we and our colleagues are also writing a cross-cultural validation study of the ESI using data obtained from several countries including India, Korea, Japan, Poland, Slovakia, Uganda, and the United States.[15] Preliminary analyses using structural equation modeling-based confirmatory factor analysis suggest that the ESI dimensions show adequate structural invariance across cultures and languages but some problems with measurement noninvariance. Specifically with the Indian sample, the ESI dimensions appear to demonstrate reasonably good factorial validity.

### Hindu religious coping scale

Tarakeshwar, Pargament, and Mahoney[16] devised the hindu religious coping scale (HRCS) to expand upon notions of religious coping identified within North American Christians; that is, the test was developed to determine how people of Hindu faith in the United States utilize religious beliefs, behaviors, and experiences to cope with adversity in life. According to the test authors, the HRCS is meant to serve as a comprehensive measure of religious coping strategies for Hindus.

Test construction started with extensive qualitative interviews of a sample of 15 Hindus. Based upon the analysis of the narratives, five functions of religious coping were identified by the test authors (i.e., to find meaning, to gain control, to gain comfort and closeness to God, to gain intimacy with others, and to achieve a life transformation) that were seen as consistent with the functions for religious coping found in American Christian samples. Next, items from existing religious coping scales that were seen as reflecting these functions were selected and revised to be more consistent with Hindu religious concepts and tenets. Where items did not exist on an available test, new items were written. An initial 23-item version of the test was piloted on a sample of 42 Hindus. After revising the test, which included the deletion of three items, the HRCS was then administered to a sample of 164 Hindus. Exploratory principal components analysis of 18 of the 20 remaining items produced three components labeled “God-Focused” Religious Coping (i.e., use of belief in God as manner of coping; God identified as both the source and solution to problems), “Spirituality-focused” Religious Coping (i.e., nontheistic use of spirituality to cope with problems such as seeking out spiritual awakening), and “Religious Guilt, Anger, and Passivity” (i.e., negative religious coping involving a nonassertive stance toward problems and/or feelings of anger or guilt arising from perception of God as being malevolent or unhelpful). In its final form, the HRCS consists of 20 items of which 18 are assigned to three subscales corresponding to the three components described earlier. The measure uses a four-point response scale ranging from 1= “haven’t been doing this at all” to 4= “have been doing this a lot” that test takers use to rate the extent to which they engage in the behavior specified by each item. Subscale scores are derived by summing responses to relevant items. Higher scores are associated with higher levels of use of religious coping strategies for any given subscale.

Psychometrically, Tarakeshwar *et al*.[16] reported satisfactory inter-item consistency coefficients for the three subscales (ranging from 0.69 to 0.85). Further, some evidence of convergent and discriminant validity through correlations within the subscales and between the subscales and demographic variables and measure of response bias (i.e., social desirability) was provided. Last, hierarchical regressions revealed that God-Focused Religious coping was a significant positive predictor of life satisfaction and Religious Guilt, Anger, and Passivity was a significant negative predictor of life satisfaction, marital satisfaction, and a significant positive predictor of depressed mood.

### Measures of hindu pathways

Tarakeshwar, Pargament, and Mahoney[17] developed the measures of hindu pathways (MHP) to provide a measure of Hindu beliefs and practices as incorporated into daily living. More specifically, the MHP is designed to provide a quantitative measure of the religious practices or “paths” that lead to the realization of Hindu religious ideals. Based upon a review of the literature, the test authors provide an overview of the major tenets of Hinduism. In so doing, they identify four main pathways which they call the path of devotion, the path of ethical action, the path of knowledge, and the path of physical restraint/yoga. The test authors also delineated various beliefs, practices, and rituals which they align with each of the pathways and completed qualitative interviews with 15 Hindus living in the United States to determine the extent to which the pathways and associated beliefs and practices are manifested in day-to-day life. Items were then generated to tap each of the religious pathways and reviewed by experts and trained students to ensure content validity. Thereafter, a preliminary version of the MHP was piloted with a sample of 42 Hindus and both items and item response formats were modified or deleted in order to improve the performance of the test. Finally, the instrument was administered to a sample of 182 Hindus to assess its psychometric properties and determine if the pathways predict psychological outcomes in a manner consistent with the research done with American Christians.

In its use with the sample of 182 Hindus, the MHP comprised four scales, each corresponding to a Hindu pathway. The Path of Devotion scale was made up of three parts; a five-item subscale tapping frequency of engagement in devotional practices (e.g., prayer, attendance to temple, performance of *puja* at home), a four item subscale assessing frequency of participation in religious festivals, and a 15-item subscale measuring frequency of performance of religious rituals. The response format for each subscale varies from a four- to six-point Likert scale. Items from the latter two subscales are summed to arrive at subscale scores. The Path of Ethical Action scale is made up of four items to which respondents use a five-point response scale ranging from 0 = never to 4 = always to indicate the extent to which they embody Hindu ethical ideals in four life domains (i.e., organization of life, work, role as a son/daughter, and personal daily habits). The Path of Knowledge scale is made up of five items. The response format varies from a four- to six-point scale depending upon the item. The Path of Physical Restraint/Yoga scale comprises four items that tap physical restraint (e.g., eating a vegetarian diet, avoidance of alcohol and tobacco) and frequency of yoga practice. Akin to two of the other scales, the response format varies across items; some items use a five-point scale, whereas the item on performing yoga uses a six-point scale.

Tarakeshwar *et al*.[17] completed a principal components analysis of the MHP using the five devotional practice items, summative scores for the festivals and ritual subscales, and the items of the remaining three scales as variables. Five components with eigenvalues in excess of 1.0 were extracted and rotated. The first component was made up of the five devotional items and the festival subscale and was consequently named Path of Devotion. One item was found to cross-load on more than two components and was subsequently dropped. The second component housed high loadings from all knowledge items and from the Rituals subscale and was labeled Path of Knowledge and Rituals. The third factor contained strong loadings from all ethical conduct items and was named Path of Ethical Action. The fourth factor had loadings from three of the physical restraint items and was dropped since it was deemed to reflect general health and dietary habits and not a Hindu path. Lastly, the fifth factor contained a high loading from the one item concerning performance of yoga as well as one item on performance of *puja*. The test authors named this component the Path of Yoga. The results of the principal components analysis were used to revise the scales such that the Path of Devotion is made up of four items and the Festivals subscale score, the Path of Knowledge and Rituals comprises the five knowledge items and the Rituals subscale score, the Path of Ethical Action is made up of four items, and the Path of Yoga made up of a single item. For the former three scales, scoring is done by summing the item responses.

The test authors report good interitem reliability coefficients for all three of the multi-item scales (alphas range from 0.76 to 0.89), evidence of score validity was also obtained, and they found the four scales to be weakly to modestly intercorrelated with each other and to be uncorrelated to social desirability.[17] In this same article, they reported analyses with demographic variables that uncovered significant correlations with age, marital status, and level of acculturation, as well as regression analyses that revealed that the one or more of the four paths served as significant predictors of life satisfaction, depressed mood, physical health, and marital satisfaction. It is noteworthy, however, that the Path of Devotion was found to be a positive predictor of depressed mood (such that greater use of this path was associated with higher depression) and a negative predictor of martial satisfaction (such that greater use of this path was associated with lower marital satisfaction), while the Path of Yoga was also found to be a negative predictor of marital satisfaction.

### Self-expansiveness level form

Friedman[18] designed the self-expansiveness level form (SELF) as one of the first paper-and-pencil instruments constructed to tap an explicitly transpersonal construct. It is an 18-item test that measures self-expansiveness or the extent to which one’s experienced sense of self corresponds to the “True Self” as defined in the spiritual, mystical, and transpersonal literature. That is, the SELF assesses self-concept expansiveness or the degree to which the self-concept is inclusive of reality as understood from a transpersonal perspective. Grounded in a spatial-temporal cartography developed as a conceptual framework, the SELF is made up of three subscales named the Personal (i.e., here and now), Transpersonal (expanded and contracted spatiality and temporality), and the Middle (i.e., those potentials of the self-concept lying between the personal and transpersonal). Respondents use a five-point Likert-type response scale to rate the extent to which they are willing to identify with the differing levels of the self-concept as delineated by the subscale items. Scoring involves numerically coding responses and then summing the items to arrive at three subscale scores.

Research with North American samples has shown that the SELF subscale scores demonstrate good reliability and adequate criterion, factorial and discriminant validity[2,3] but somewhat weaker convergent validity.[19] With an Indian sample, the SELF has been found to produce somewhat less reliable scores and factor analysis of the SELF Personal and Transpersonal items with data obtained from this Indian sample generated partial support for the factorial validity of the test with the Personal subscale demonstrating greater robustness than the transpersonal subscale.[20]

### Spiritual orientation inventory

Elkins, Hedstrom, Hughes, Leaf, and Saunders[21] developed the spiritual orientation inventory (SOI) to serve as a nonreligious measure of spirituality, constructing the instrument based upon a content analysis of literature from a variety of seminal thinkers and writers in the humanistic, existential, and depth psychological traditions. Emerging from their content analysis, the test authors identified nine components that comprise spirituality: Transcendent Dimension (i.e., spiritual experience), Meaning and Purpose in Life, Mission in Life (i.e., a sense of vocation arising from one’s sense of spirituality), Sacredness in Life (i.e., belief that life is sacred), Material Values (i.e., recognition that fulfillment cannot be derived from material objects), Altruism (i.e., compassion), Idealism (i.e., a sense of vision and commitment to the improvement of the world), Awareness of the Tragic (i.e., recognition of the suffering inherent in the human condition), and Fruits of Spirituality (i.e., effect of spirituality on the quality of one’s life and relationships to others, including a higher power or powers).

Initially, Elkins *et al*.[21] constructed a pool of 200 items to operationalize each of the nine aforementioned components. Items were thereafter eliminated if (a) they did not demonstrate content validity as determined by a panel of experts and (b) they did not discriminate between people identified as highly spirituality versus those considered low in spirituality. This left a final pool of 85 items that are unevenly distributed across nine subscales. The 85-item version of the SOI utilizes a seven-point response scale ranging from 1 = strongly disagree to 7 = strongly agree with which respondents use to rate the extent to which they agree with the content of each of the items. Although most of the items are positively worded, a number of them are negatively worded. Consequently, scoring of the instrument first requires reverse coding negatively worded items before summing the item responses to arrive at nine subscale scores. A total SOI score could be obtained by simply summing the nine subscale scores.

Available research suggests that with North American samples, the SOI produces scores of adequate reliability and validity.[2] Of interest to its cross-cultural properties, Zainuddin[22] used data obtained from a sample of 219 Indian Muslim Teachers to factor analyze the SOI subscales. Results suggest that the SOI demonstrates a two-factor structure wherein one factor is an experiential factor and the second is a values factor. These results are similar to a factor analytic study done by MacDonald[13] where the SOI subscales, along with 10 other measures of spirituality and associated constructs, were found to contribute to factors related to spiritual experience, spiritual beliefs, and the products of spirituality with data obtained from a sample of 534 Canadian university students.

### Spiritual transcendence scale

Piedmont[23] developed the spiritual transcendence scale (STS) to serve as a measure of spirituality or, more specifically, spiritual transcendence. This construct was considered by Piedmont to reflect a broad motivational domain of psychological functioning: “Transcendence is a fundamental capacity of the individual, a source of intrinsic motivation that drives, directs, and selects behaviors”[23] (p. 988). Within the domain, and based upon both literature and focus groups constituted of religious experts who discussed the nature of transcendence from the perspective of different faith systems, Piedmont reports several components or facets to spiritual transcendence, including a sense of connectedness, universality, prayer fulfillment, tolerance of paradoxes, nonjudgmentality, existentiality, and gratefulness. Piedmont initially created a pool of 65 items that he felt represented the facets of transcendence. Thereafter, using an iterative factor analytic process, he systematically eliminated items that did not differentiate spiritual transcendence from personality variables. This resulted in the exclusion of 41 items which reflected several of the seven facets. In its current final form, the STS is a 24-item test that is made up of three subscales called Connectedness, Prayer Fulfillment, and Universality. Connectedness is defined as “a sense of personal responsibility to others that is both vertical, cross-generational commitments, and horizontal, commitments to others in [the] community” (p. 996). Prayer Fulfillment is described as “an experienced feeling of joy and contentment that results from prayer. Prayer provides a sense of personal strength. Prayer is consuming and orients one to another state of being (p. 995). Finally, Universality is referred to as “a belief in the unity and purpose of life; a feeling that all life is interconnected and a sense of a shared responsibility of one creature to another” (p. 995). Respondents are provided a five-point response scale ranging from 1= strongly agree to 5= strongly disagree to rate how much they agree with the item as being applicable to themselves. In addition to a self-report version of the test, Piedmont also developed a parallel peer-rating form. The only difference between the two measures is that the items for the later version are written in the third as opposed to first person. Scores for the subscales are derived by summing item scores. A total STS score can be generated by summing all item scores.

Data on the psychometric properties of the STS with American samples suggest that scores from both the self-report and peer-report versions of the test are adequately reliable (e.g., alphas ranging from 0.64 to 0.91 across all scales and both tests) and, as importantly, there is evidence supporting the validity of scores with American respondents (e.g., Piedmont found through both exploratory and confirmatory factor analysis that the STS items clearly fit a three-factor structure and demonstrated convergent and discriminant validity, criterion validity, and incremental validity [i.e., the ability to predict the psychosocial outcomes listed and beyond personality variables]).[23,24] Outside of American samples, the STS has not fared as well, though it still holds promise; using a sample of 273 Indian students, of which 218 were Hindu, 87 Christian, and 64 Muslim, Piedmont and Leach[25] found the STS to have marginal inter-item consistency (e.g., for the total sample, alpha coefficients ranged between 0.23 and 0.67 for the subscales; alpha was 0.71 for the total scale). A confirmatory factor analysis of STS items found only partial support for instrument test of the three-factor structure resulted in inconsistent fit statistics. Deletion of the Connectedness subscale items resulted in an improvement in the model fit. Piedmont and Leach concluded that this provides supportive evidence for the remaining two STS subscales. On a more positive note, predictive and incremental validity was found to be comparable to that observed with American samples.

### Vedic personality inventory

Wolf[26] developed the vedic personality inventory (VPI) was to test the validity of the Vedic concept of the three *gunas* (i.e., modes of nature; *sattva, rajas, tamas*) and to determine its utility as a psychological typology. Using the Vedic literature as the basis for conceptual development and item construction, the initial form the test consisted of 90 items divided across three subscales corresponding to the three gunas and employed a seven-point Likert scale response format. Based upon an item-level factor analysis with a sample of 247 Americans, 10 items were dropped from the test, leaving a total of 80 items. Reliability analyses suggest the VPI has good interitem consistency (alphas in excess of .85 for all three scales). In addition, Wolf produced evidence of convergent and discriminant validity. Further psychometric work done by Wolf[27] with a sample of 619 Americans resulted in the test being shortened to 56 items. Reliability of the subscales in the shortened test continued to be satisfactory (α > 0.90). Moreover, Wolf found further evidence of construct validity as manifest in theoretically expected correlations with conceptually similar and dissimilar measures. Additional psychometric support for the VPI was generated by Stempel, Cheston, Greer, and Gillespie[28] who found the scales of the VPI to correlate in a theoretically expected manner with measures of daily spiritual experiences and psychopathology.

## RECOMMENDATIONS FOR RESEARCH

Although it may be helpful for the reader to know of the existence of measures that may be effective for use in yoga (and, more generally, spiritual/transpersonal) research, to simply give information on available tests related to spiritual and transpersonal constructs is not sufficient. Despite the fact that there are well more than 100 instruments in the published literature, and many more if we include explicit measures of religiousness, we have noted that there is little convergence of test use across studies and, by association, little coherence or cumulativeness of knowledge garnered through spiritual and transpersonal research.[29] Stated differently, few of the extant measures have been used in a programmatic manner across studies or even across investigators, thus resulting in a disorganized and difficult-to-decipher body of research. Consequently, we believe it important to provide concrete recommendations for how spiritual and transpersonal research involving tests should proceed.

(1) Within a single study, use multidimensional tests and/or multiple measures of a construct whenever possible. Since most concepts and phenomena in spiritual and transpersonal psychologies are complex, unidimensional instruments that assess these constructs as a global entity are really not sufficient for most research purposes. If an investigator is going to use tests in a study, then consideration should be given to using multidimensional scales and/or multiple measures. Ideally, psychometric tests should be combined with other operational measures (e.g., behavioral observation, structured or semi-structured interviews) so that studies utilize a multimethod approach to data gathering for any given construct. This helps to reduce problems associated with shared method variance.

(2) Thoroughly validate measures before employing them for other research purposes. This is an extremely important recommendation. None of the tests presented in this article have been, in our opinion, sufficiently validated to warrant strong confidence in their use with respondents from varying populations. The validity and reliability of a test and the scores it generates is never finalized. Rather, knowledge about the psychometrics is based upon a cumulative body of work and what has been found with past samples may or may not generalize to new samples, especially if the new samples are drawn from a population not previously studied. As such, there is an ongoing need to check the reliability and validity of a test with all samples. If a study incorporates a multi-method approach to measurement, this builds into the study opportunities to gather data that will allow for an evaluation of a measure (primarily in the form of convergent validity).

Nested within this recommendation is the issue of universalism versus particularism. As has been argued by Moberg,[30] it may not be appropriate to assume that a spiritual or transpersonal construct is universally meaningful or applicable to all human beings. We also have pointed out previously that differences arising from culture, language, and religion need to be considered in generating and interpreting data about the validity and utility of a test.[31]

(3) Try to use the same instruments and data gathering methods across studies. As we have said elsewhere, “research traditions and subdisciplines are built and maintained through the ongoing use of a theory and/or methodology in exploring a phenomen[on]/behavior”[1] (p. 122).

## A PROGRAM OF RESEARCH

**Figure 1:**
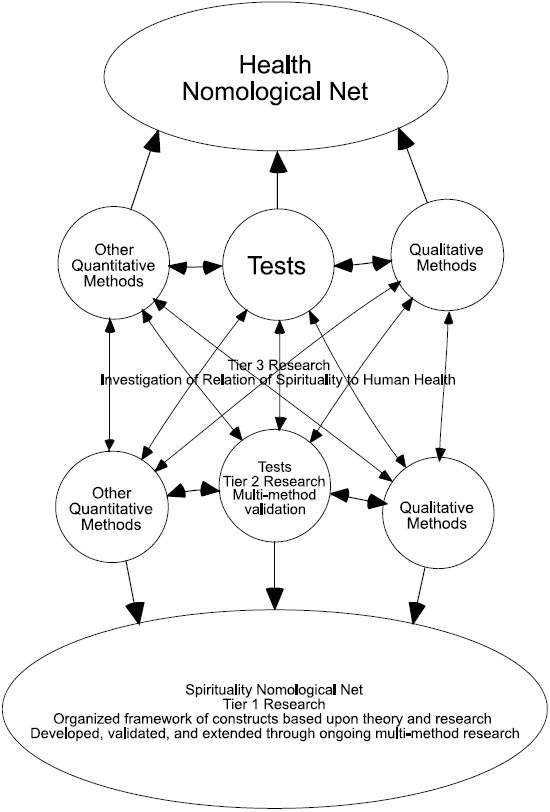
Graphic depiction of proposed research program using spirituality and health as examples

In essence, these recommendations can be consolidated into a general multilayered and open-ended program of research that takes into consideration the complexities of the phenomena of interest, while bringing transpersonal science more into alignment with the manner in which mainstream psychological science works. Figure 1 provides a graphic representation of the research program using spirituality as an example.

At the first and most substantive tier, systematic qualitative (e.g., interviews, literature reviews) and quantitative (e.g., tests, meta-analyses), research is needed to identify the features of human experience and functioning that comprise whatever it is that an investigator considers spiritual or transpersonal, whether a concept or a phenomenon (e.g., what is spirituality? What is meditation? What is wisdom? What is transpersonal experience? What is personal transformation?). This will involve the delineation of inclusionary and exclusionary criteria through which concepts, theory, and research are evaluated to determine if they should be considered part of the transpersonal domain. Researchers need to identify a set of constructs—preferably ones that cannot be simply reduced to another domain of human functioning (e.g., personality, psychopathology) but can still be understood in terms of individual differences that can be used as the basis for the development of specialized theories and research designs. That is, it is of the utmost importance to create a nomological net of constructs and theories[32] from which a cumulative and organized body of knowledge can emerge. Embedded within this tier is the need for studies that establish the incremental validity and explanatory power of transpersonal theory and methodologies for the understanding human functioning above and beyond what mainstream psychological theory and science provide.

Existing psychometric measures provide a terrific starting approximation of the parameters of many key constructs and phenomena which can be rigorously evaluated through conventional empirical methodologies and ultimately used to establish a nomological net. In the case of many available measures, the test authors have already given fairly thorough consideration to the content of their concepts of interest while developing their instruments using quantitative and/or qualitative methods. Accordingly, what needs to be done, and done on an ongoing basis, is scientific investigation into the convergence of these differing measures and their underlying conceptual models so as to ensure that they are, in fact, measuring relevant and recognized components and features of the construct domain. Such studies are desperately needed across cultures and languages as well as across the lifespan, since little has been done to evaluate the universality and equivalence of constructs for different ethnic and linguistic groups and for people at different ages and stages of life. Ideally, quantitative empirical investigations will be balanced with qualitative studies (e.g., focus groups, interviews, psychohistorical and phenomenological analyses) that seek to confirm the “fit” of the construct to a person’s beliefs and experience.

As an integral part of defining the nomological net, the second important tier of the proposed research program involves giving considerable attention to the critical and rigorous examination of the psychometric properties of available measures, including investigations into their validity and utility with different cultural and developmental groups. As deficiencies and limitations of tests are identified, especially as they relate to their underlying theoretical bases, researchers should develop revised models and measurement instruments which, in turn, can be the focus of further study.

As the nomological net is created, replicated and extended, the third tier of the research program comes into effect. Core constructs (i.e., concepts whose nature and empirical relations have been robustly supported in both theory and research) should serve as the basis of study into the relation of spiritual and transpersonal theory to other domains of human functioning including well-being, health, psychopathology, social functioning, personality, intelligence, and so forth.

Although research can be done at the level of one or more of the tiers concurrently, third tier research really should only start after sufficient and verifiable knowledge about the nature and validity of the transpersonal construct that we want to study has been obtained. To proceed without such knowledge is to run the risk of perpetuating current practices and deepening the confusion about the veracity and significance of spiritual and transpersonal constructs for psychological science.

## CONCLUSION

We hope that the information presented in this article serves as a catalyst for new avenues of spiritual and transpersonal research in the area of yoga studies. Should readers be interested in pursuing the proposed research program, there are several identifiable constructs domains that appear to us as ready for development. Measures reported in this article have been selected with programmatic research possibilities in mind. These construct domains include (a) methods of transformation, including different forms of practice whose goals are spiritual and transpersonal in nature, such as meditation, yoga, prayer, devotion, service (Measure of Hindu Pathways), (b) modes and states of consciousness (ASASC, Phenomenology of Consciousness Inventory, Boundary Questionnaire, Ego Permissiveness Inventory, Transliminality Scale), (c) spirituality (ESI, STS, SOI, Psychomatrix Spirituality Inventory), (d) modes and states of selfhood (e.g., SELF, VPI, Temperament and Character Inventory, Feelings, Reactions, Beliefs Survey), and (e) health and well-being (Scales of Psychological Well-Being, Perceived Wellness Survey).
